# Glycan degradation promotes macroautophagy

**DOI:** 10.1073/pnas.2111506119

**Published:** 2022-06-22

**Authors:** Alice D. Baudot, Victoria M.-Y. Wang, Josh D. Leach, Jim O’Prey, Jaclyn S. Long, Viola Paulus-Hock, Sergio Lilla, David M. Thomson, John Greenhorn, Farah Ghaffar, Colin Nixon, Miep H. Helfrich, Douglas Strathdee, Judith Pratt, Francesco Marchesi, Sara Zanivan, Kevin M. Ryan

**Affiliations:** ^a^Tumour Cell Death and Autophagy Laboratory, Cancer Research UK Beatson Institute, Glasgow G61 1BD, United Kingdom;; ^b^School of Veterinary Medicine, College of Medical, Veterinary, and Life Sciences, University of Glasgow, Glasgow G61 1QH, United Kingdom;; ^c^Strathclyde Institute of Pharmacy and Biomedical Science, University of Strathclyde, Glasgow G4 0RE, United Kingdom;; ^d^Institute of Medical Sciences, University of Aberdeen, Aberdeen AB25 2ZD, United Kingdom;; ^e^Institute of Cancer Sciences, University of Glasgow, Glasgow G61 1QH, United Kingdom

**Keywords:** macroautophagy, α-l-fucosidase 1, lysosomes, fucosidosis

## Abstract

Macroautophagy promotes cellular homeostasis by delivering cytoplasmic constituents to lysosomes for degradation [Mizushima, *Nat. Cell Biol.* 20, 521–527 (2018)]. However, while most studies have focused on the mechanisms of protein degradation during this process, we report here that macroautophagy also depends on glycan degradation via the glycosidase, α-l-fucosidase 1 (FUCA1), which removes fucose from glycans. We show that cells lacking FUCA1 accumulate lysosomal glycans, which is associated with impaired autophagic flux. Moreover, in a mouse model of fucosidosis—a disease characterized by inactivating mutations in *FUCA1* [Stepien *et al.*, *Genes (Basel)* 11, E1383 (2020)]—glycan and autophagosome/autolysosome accumulation accompanies tissue destruction. Mechanistically, using lectin capture and mass spectrometry, we identified several lysosomal enzymes with altered fucosylation in FUCA1-null cells. Moreover, we show that the activity of some of these enzymes in the absence of FUCA1 can no longer be induced upon autophagy stimulation, causing retardation of autophagic flux, which involves impaired autophagosome–lysosome fusion. These findings therefore show that dysregulated glycan degradation leads to defective autophagy, which is likely a contributing factor in the etiology of fucosidosis.

## Significance

Macroautophagy preserves cellular integrity and, as a result, protects against various forms of human disease. It is therefore imperative that we understand how the process is controlled. Our finding that appropriate glycan degradation is an important and distinct control point for successful macroautophagy represents a fundamental insight into this process. Furthermore, our finding that fucosidosis, a congenital disorder of glycosylation, involves impairment of macroautophagy opens up new possibilities for treating this currently incurable disease.

Maintaining the fidelity of cellular constituents is critical for cell viability and organismal health. Autophagy (literally “self-eating”) is a group of catabolic processes that deliver cytoplasmic constituents to lysosomes for degradation (1). The best-studied form of autophagy is macroautophagy, which is characterized by specialized organelles called autophagosomes that facilitate the delivery of cargoes destined for degradation (1).

Macroautophagy is initiated by the formation of a membranous structure termed an isolation membrane, which forms from a variety of sources within the cell (2–5). Two ubiquitin-like conjugation systems then grow this membrane via the action of evolutionarily conserved autophagy-related (ATG) proteins. The growing double membrane finally fuses to form the ball-like autophagosome, which encapsulates cargoes including damaged/misfolded proteins and organelles. Autophagosomes can then fuse with endosomes to form amphisomes, but ultimately fusion occurs with lysosomes to form autolysosomes, within which the cargo of the autophagosome is degraded by lysosomal hydrolases. Finally, the degraded constituent parts—including amino acids, lipids, sugars, and minerals—are then recycled into the cytoplasm, where they are used in biosynthetic pathways or, in some cases, further catabolized to generate ATP (6). Since macroautophagy is a major mechanism for the degradation of long-lived proteins and the only mechanism to degrade organelles described so far, perturbation of autophagy can lead to a variety of diseases, including neurodegenerative diseases, inflammatory diseases, diabetes, and cancer (7).

Glycosylation is essential for the correct functioning of cellular machinery. The importance of this process is exemplified by the fact that ∼50% of all proteins are glycosylated and over 50 diseases involve deregulated glycosylation (8, 9). Glycosylation of proteins is complex but occurs via two major pathways: *N*-linked glycosylation, in which glycans are attached to asparagine residues, and *O*-linked glycosylation, in which glycans are attached to either serine or threonine. In both types, glycans are attached to proteins by glycosyltransferases and, conversely, glycans are removed by glycosidases.

**Fig. 1.**
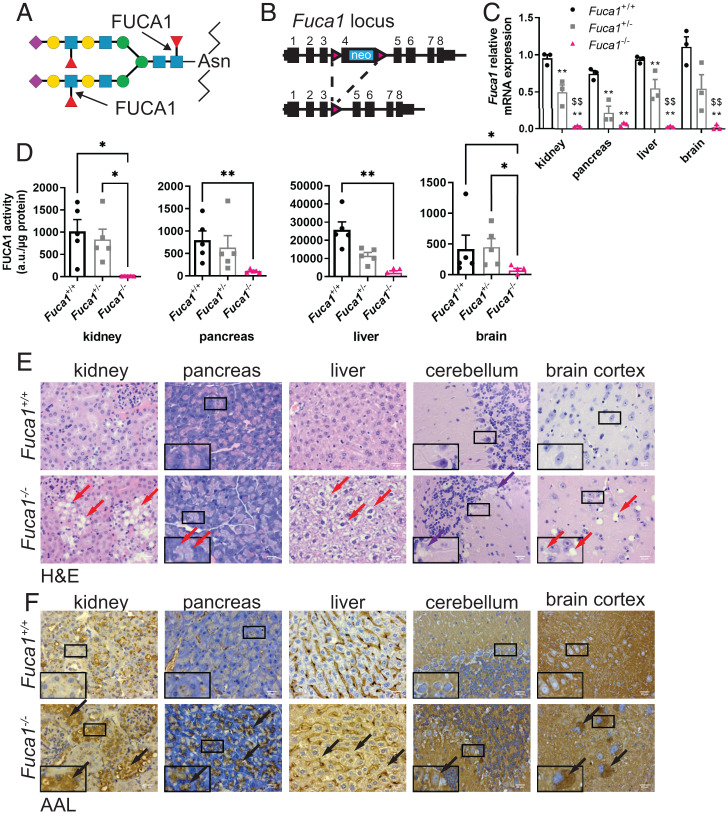
Generation of a mouse model of fucosidosis. (*A*) Schematic showing that FUCA1 cleaves both terminal fucose and core fucose linked to an *N*-acetylglucosamine (purple diamond: *N*-acetylneuraminic acid; yellow circle: galactose; blue square: *N*-acetylglucosamine; green circle: mannose; red triangle: fucose). (*B*) Schematic representation of the *Fuca1*-targeted allele and the mutated allele after Cre recombination. (*C*) Relative *Fuca1* mRNA expression from different organs was determined by qRT-PCR (three mice per genotype). Results are represented as mean ± SEM (two-way ANOVA with Bonferroni correction. *Fuca1*^+/+^ vs. *Fuca1*^+/−^ or *Fuca1*^−/−^ **P < 0.01. *Fuca1*^+/−^ vs. *Fuca1*^−/− $$^*P* < 0.01). (*D*) FUCA1 activity was measured from lysates of the indicated organs (five mice per genotype, mean ± SEM) and expressed as arbitrary units (a.u.) per microgram of protein (Kruskal–Wallis test; *P < 0.05, **P < 0.01). (*E* and *F*) Organ sections of both *Fuca1*^+/+^ (*Upper*) and *Fuca1*^−^*^/−^* (*Lower*) mice were stained with H&E (*E*) and AAL (*F*). Red arrows indicate cytoplasmic vacuolation, purple arrows indicate Purkinje cell loss, black arrows indicate accumulation of glycan species in the specified tissues. Pictures were taken using a Zeiss AX10 microscope with a 40× objective. The images shown are representative of changes observed in six mice (aged between 90 and 220 d). (Scale bars, 20 µm.) *Insets* are magnified (2×) crops from the same images to show specific staining.

The lysosomal enzyme α-l-fucosidase 1 (FUCA1, EC 3.2.1.51) is critical for the degradation of *N*-linked glycans. FUCA1 cleaves fucose from both the terminal branches of glycans, as well as the moiety known as “core fucose” on *N*-glycans, which is linked to the *N*-acetylglucosamine that is attached to asparagine in the peptide backbone (Fig. 1*A*) (10–13). Cleavage of core fucose is an essential event for further degradation of the glycan, and therefore FUCA1 is critically important for the breakdown of the glycan component of *N*-linked glycoproteins (12, 14).

The importance of *FUCA1* is exemplified by mutations in the gene, which lead to the congenital lysosomal storage disorder fucosidosis (15). Individuals carrying two mutated alleles of *FUCA1* often have no fucosidase activity and have lysosomal accumulation of glycans in multiple tissues. As a result, fucosidosis patients develop multiple pathologies, including neurodegeneration, growth retardation, impaired immunity, and visceromegaly. The majority of patients with fucosidosis die before 30 y of age (16, 17).

Due to the large proportion of proteins that are glycosylated, we postulated that macroautophagy must be dependent not only on enzymes that degrade polypeptides, but also on those that degrade glycans. To test this, we generated mice that are deficient in FUCA1 and examined the impact on macroautophagy. These experiments clearly revealed that fucosidase activity promotes both autophagosome–lysosome fusion as well as the turnover stage of macroautophagy, and that modulation of autophagy has a significant impact on the accumulation of glycans associated with fucosidosis.

## Results

### Generation and Phenotypic Analysis of *Fuca1*^−/−^ Mice.

To understand the consequences of deregulated glycosylation on autophagy, we generated mice that are deficient in FUCA1. To do this, we designed a conditional knockout (KO) allele for *Fuca1* (*Fuca1*^flox/flox^) in which exon 4 of *Fuca1* is flanked by two loxP sites (Fig. 1*B*). Mice that were either wild-type or contained one or two copies of the engineered allele were then crossed to a whole-body deleter Cre recombinase (delCre) to generate animals that are either wild-type, hemizygous, or null for *Fuca1*.

To test the impact of *Fuca1* deletion, we first examined *Fuca1* mRNA levels in a variety of tissues by qRT-PCR. This clearly showed that delCre *Fuca1*^flox/flox^ mice had either very low or undetectable levels of *Fuca1* mRNA, whereas delCre *Fuca1*^flox/+^ mice had ∼50% expression when compared to controls (Fig. 1*C*). The effect on fucosidase activity was also measured using a previously described enzymatic assay (18). This assay revealed that *Fuca1*-null animals had extremely low levels of FUCA1 activity in multiple tissues (Fig. 1*D*). Interestingly, while the livers of animals that were *Fuca1* hemizygous had an ∼50% decrease in FUCA1 activity when compared to wild-type littermates, hemizygosity in *Fuca1* did not have a significant effect on FUCA1 activity in kidney, pancreas, and brain (Fig. 1*D*), suggesting either increased FUCA1 protein abundance or enzymatic activity in these tissues.

### Loss of FUCA1 Causes Tissue Destruction and Accumulation of Glycans.

We next examined if loss of FUCA1 was associated with changes in tissue architecture. Consistent with previous studies on fucosidosis (19, 20), histological analysis revealed prominent cytoplasmic vacuolation in a variety of tissues with marked effects in neurons and glia in the central nervous system, hepatocytes in the liver, acinar cells in the pancreas, and the kidney (Fig. 1*E*). We also observed loss of Purkinje cells in the cerebellum of *Fuca1*-null animals (Fig. 1*E*).

Since FUCA1 is a glycosidase, we performed further histological analyses to determine if the vacuolation observed in multiple tissues was associated with accumulation of glycans. To test this, we utilized two fucose-binding lectins: *Aleuria aurantia* lectin (AAL), which recognizes fucose in multiple configurations (21), and *Ulex europaeus* agglutinin I (UEAI), which preferentially binds to fucose-linked α-1,2 to galactose (21–23). Using these lectins, we found that the accumulation of glycan species containing fucose in a number of different configurations was widespread in tissues lacking FUCA1 (Fig. 1*F*). In contrast, accumulation of fucose-linked α-1,2 to galactose was only markedly evident in the pancreas (*SI Appendix*, Fig. S1). In summary, loss of FUCA1 causes changes in multiple tissues that are associated with widespread, but also selective accumulation of glycan species.

Since fucosidosis patients have decreased life expectancy (17, 24, 25), we examined the viability of *Fuca1*-null and *Fuca1* hemizygous mice over time. While *Fuca1* hemizygous mice had lifespans equivalent to those of wild-type animals, *Fuca1*-null mice exhibited progressive deterioration and had to be culled between 200 and 350 d after birth (*SI Appendix*, Fig. S2*A*).

At birth, mice from all genotypes appeared physically normal and had equivalent weights, but over time *Fuca1*-null mice gained weight more quickly than their wild-type counterparts, with hemizygotes having an intermediate phenotype (*SI Appendix*, Fig. S2*B*). This effect was more pronounced in male animals (*SI Appendix*, Fig. S2*B*). We next examined whether the weight gain was associated with organomegaly as this is one characteristic of individuals with fucosidosis, and we found that male *Fuca1*-null mice had increased liver weights when compared with wild-type mice (*SI Appendix*, Fig. S2*C*).

Just like during brain-specific loss of autophagy (26), neurodegeneration and perturbed motor activity are key characteristics of fucosidosis (17, 24, 25, 27). We noticed that aged *Fuca1*-null mice were less motile than littermate controls. To examine this further, we performed a SHIRPA (SmithKline Beecham, Harwell, Imperial College, Royal London Hospital, phenotype assessment) behavioral test battery. These experiments revealed that *Fuca1*-null mice appear equivalent to wild-type animals in many aspects of physical and neurological health (*SI Appendix*, Table S1). However, in more detailed assays of motor function, it was clear that *Fuca1*-deficient mice were significantly impaired. Analysis of performance in the hanging wire test indicated that FUCA1 deficiency has an impact on grip strength. Again, males seemed to be more affected than females, with a deficiency in the hanging wire test being evident even in male mice hemizygous for *Fuca1* (*SI Appendix*, Fig. S2*D*). Taken together these results suggest that *Fuca1*-null mice have either a reduced signal to the musculature, reduced muscular strength, or a reduced aversion to the fall distance that the hanging wire imposes.

Mice were next examined in the open-field test to assess spontaneous locomotor activity and habituation, which can be impaired in a range of conditions, including aging and neurodegeneration. In these tests, wild-type and hemizygous mice showed the typical reduction in distance traveled over time due to habituation (*SI Appendix*, Fig. S2*E*). The *Fuca1*-null mice, however, did not show any elevated locomotor activity in the early stages of testing compared to the wild-type and hemizygous mice; in fact, the *Fuca1*-null mice showed no variation in locomotor activity across the entire 30-min testing period. This was true for both males and females. Univariate analysis restricted to the first 5-min time bin revealed a significant effect of genotype (*F*_2,__50_ = 12.00; *P* = 0.0001). Post hoc analysis with Bonferroni correction showed this effect to be exclusively related to the homozygotes: homozygotes vs. wild-types *P* = 0.001; homozygotes vs. heterozygotes *P* = 0.0001; wild-types vs. heterozygotes *P* = 1.00. The reduction in distance traveled in the open-field test was also reflected in overall velocities upon introduction into a new environment (*SI Appendix*, Fig. S2*F*), suggesting that the deficit is related to overall motor output.

### Loss of FUCA1 Results in Perturbed Autophagy.

**Fig. 2.**
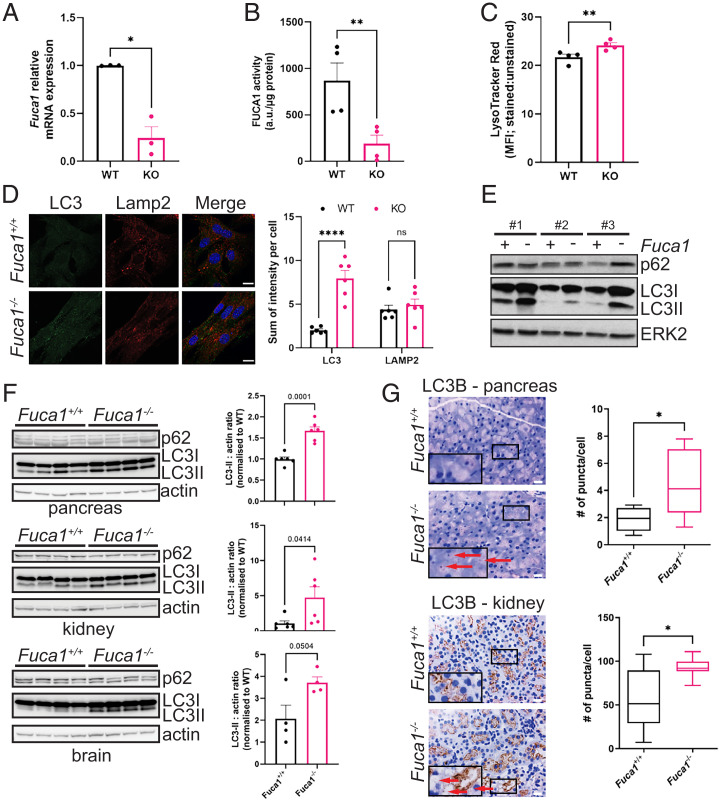
FUCA1 loss modulates autophagy both in vitro and in vivo. (*A*) Primary *Fuca1*^flox/flox^ MEFs were infected with a retrovirus expressing the Cre recombinase (KO) or a retrovirus control empty vector (WT). *Fuca1* relative mRNA expression was determined by qRT-PCR and expressed as the mean of three independent experiments ± SEM (two-tailed paired *t* test *P = 0.0238). (*B*) FUCA1 activity was measured and expressed as the mean of three independent experiments ± SEM (two-tailed paired *t* test **P = 0.0073). (*C*) LysoTracker red staining was analyzed by flow cytometry and expressed as the mean fluorescence intensity (MFI) ratio of stained versus unstained samples (four independent experiments ± SEM; two-tailed paired *t* test **P = 0.0046). (*D*) *Fuca1*^flox/flox^ CTRL (WT) and *Fuca1*^flox/flox^ CRE (KO) MEFs were analyzed by immunofluorescence using antibodies directed against LC3B and Lamp2. Nuclei were stained with DAPI. Staining was quantified from six different pictures and expressed as the sum of intensity per cell ± SEM (two-way ANOVA, Sidak’s multiple comparison test: LC3 ****P < 0.0001; LAMP2 n.s.). (Scale bar, 20 µm.) (*E*) Primary *Fuca1*^flox/flox^ MEFs isolated from three different embryos were infected with a Cre recombinase retrovirus (*Fuca1*-) or a retrovirus control empty vector (*Fuca1*+). LC3B and p62 expression were assessed by immunoblot. Total ERK was used as a loading control. (*F*) Pancreas, kidney, and brain samples from *Fuca1*^+/+^ and *Fuca1*^−/−^ mice were lysed, and LC3B and p62 analyzed by immunoblot. Representative immunoblot obtained from four mice per genotype is shown. Actin was used as a loading control. LC3II : actin ratio was quantified by densitometry using four to six different samples per genotype (two-tailed unpaired *t* test; pancreas ***P = 0.0001, kidney *P = 0.0414, brain *P* = 0.0504). (*G*) LC3B IHC of pancreas and kidney of *Fuca1*^+/+^ and *Fuca1*^−/−^ mice. LC3^+^ puncta in pictures (red arrows) were quantified for *Fuca1*^+/+^ pancreas (*n* = 6) and kidney (*n* = 10), and *Fuca1*^−/−^ pancreas (*n* = 6) and kidney (*n* = 9). (Scale bar, 20 µm.) *Insets* are magnified (2×) crops from the same images to show specific staining. Box plots represent the quantification of LC3 puncta per cell (Mann–Whitney test; pancreas *P = 0.026, kidney *P = 0.0124).

With a view to understanding how loss of FUCA1 mechanistically affects cellular physiology and organismal health, we isolated *Fuca1*^flox/flox^ mouse embryonic fibroblasts (MEFs) for study in vitro. These cells were infected with either a retrovirus expressing Cre recombinase to delete *Fuca1* or an empty control retroviral vector. Loss of FUCA1 was confirmed by qRT-PCR (Fig. 2*A*) and FUCA1 enzymatic assay (Fig. 2*B*). Since FUCA1 is a lysosomal enzyme, we first stained cells with LysoTracker red (which stains all acidic compartments) for flow cytometry and this revealed a small but significant increase in the abundance of these compartments upon loss of FUCA1 (Fig. 2*C*). In addition, since macroautophagy is intrinsically dependent on lysosomal function (28), we also examined if loss of FUCA1 had an impact on this process. In the first instance we stained MEFs with antibodies against the lysosomal membrane protein Lamp2 and the autophagosome membrane protein LC3 (both of which are also present in autolysosomes). In cells with low levels of autophagosomes, the majority of LC3 is in a form termed LC3-I, which is diffusely localized within the cytoplasm. Upon formation of autophagosomes, LC3-I is cleaved and conjugated to phosphatidylethanolamine. In this form, termed LC3-II, LC3 becomes anchored into the autophagosome membrane via its lipid moiety, and its presence as distinct puncta within cells can be used to detect and quantify autophagosomes (29). These immunofluorescence experiments revealed no significant change in the levels of Lamp2 between cells, but loss of FUCA1 caused a considerable increase in the number of LC3^+^ puncta per cell, indicating accumulation of autophagosomes (Fig. 2*D*). To confirm this result, we analyzed LC3 by Western blotting in three independent MEF culture isolates. Using an antibody that can detect the mobility shift caused by the cleavage and lipidation of LC3, this again showed that *Fuca1*-null cells have higher levels of LC3-II and, by inference, higher levels of autophagosomes (Fig. 2*E*). In contrast, accumulation of the selective autophagy adaptor p62 was not so pronounced and more variable (Fig. 2*E*).

To determine whether the increased number of autophagosomes was also a consequence of loss of FUCA1 in vivo, we examined protein lysates from the pancreas, kidney, and brain of wild-type and *Fuca1*-null mice. This analysis again revealed a marked increase in the levels of LC3-II in the absence of FUCA1 (Fig. 2*F*). Again, we did not observe accumulation of p62 in the soluble fraction of lysates from *Fuca1*-null tissues. We also stained tissue from the pancreas and kidney for LC3, which showed increased levels of LC3 puncta in *Fuca1*-null mice (Fig. 2*G*). Expression of LC3 in the brain, as assessed by immunohistochemistry, is high under homeostatic conditions, making it difficult to distinguish changes in LC3 puncta in wild-type versus *Fuca1*-null mice. Instead, we stained brain tissues with the mitochondrial marker VDAC and observed an accumulation of mitochondria in multiple regions of *Fuca1*-null brains (*SI Appendix*, Fig. S3). Since mitochondria in the brain are normally turned over via autophagy, this suggests that FUCA1 has a role in autophagy in multiple organs.

Furthermore, to test if FUCA1 loss leads to a general endo-lysosomal defect, we incubated wild-type and *Fuca1*-null MEFs with two fluorescently labeled substrates that can be used to track endocytosis (dextran) and lysosomal protease activity (DQ-BSA). Both substrates are taken up by endocytosis, but DQ-BSA fluorescence is quenched until the heavily labeled BSA reaches the lysosomes, where proteases break down the BSA, dequenching the fluorescence. Fluorescent dextran, on the other hand, fluoresces all the way through the endocytic pathway and can therefore be used to measure endocytic uptake and uptake rates. This time-course experiment revealed that wild-type and *Fuca1*-null MEFs have comparable endocytic uptake rates as well as lysosomal activity against DQ-BSA (*SI Appendix*, Fig. S4*A*), indicating that loss of FUCA1 does not lead to a general defect in the delivery or degradation of all cargoes in the lysosome.

### Loss of FUCA1 Impairs Autophagic Flux.

**Fig. 3.**
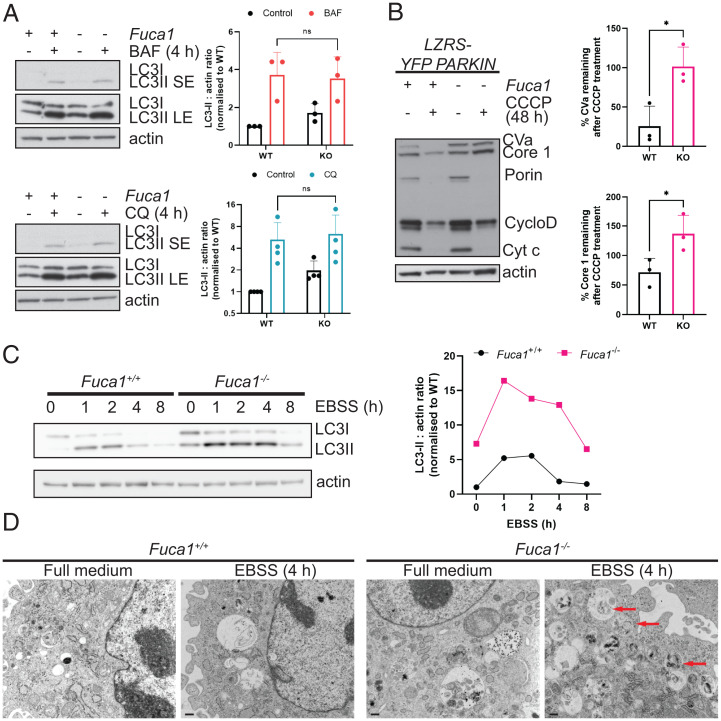
FUCA1 loss impairs autophagy flux. (*A*) Immortalized *Fuca1*^flox/flox^ MEFs were infected with a retrovirus expressing the Cre recombinase (KO) or a retrovirus control empty vector (WT) and treated with 100 nM BAF or 10 µM CQ for 4 h. Cell lysates were analyzed by immunoblotting using antibodies directed against LC3B and actin, which was used as a loading control. The immunoblot shown is representative of the results observed in three to four independent experiments, and quantified (*Right*) ± SEM (two-way ANOVA, Sidak’s multiple comparison test: n.s.). LE, long exposure; SE, short exposure. (*B*) *Fuca1*^+/+^ and *Fuca1*^−/−^ MEFs were infected with a retrovirus expressing YFP-Parkin (LZRS-YFP-PARKIN). Cells were then treated with 12.5 μM CCCP for 48 h. Cell lysates were analyzed using the MitoProfile Membrane integrity WB Antibody Mix. Actin was used as a loading control. The immunoblot shown is representative of the results observed in three independent experiments. The percentage of remaining CVa and Core 1 in *Fuca1* WT and KO MEFs upon CCCP treatment is quantified ± SEM (two-tailed unpaired *t* test, CVa *P = 0.021, Core 1 *P = 0.0445). (*C*) Immortalized *Fuca1*^+/+^ and *Fuca1*^−/−^ MEFs were incubated in EBSS for the indicated time period. Cell lysates were analyzed by Western blot using LC3B and actin antibodies. The LC3II : actin ratio is plotted on the right. (*D*) Primary *Fuca1*^+/+^ and *Fuca1*^−/−^ MEFs were incubated in EBSS for 4 h. Electron microscopy images show autophagic degradative vacuoles (red arrows). (Scale bars, 500 nm.)

Macroautophagy is a dynamic process during which autophagosomes represent a transient midpoint. Accumulation of autophagosomes can therefore indicate an increase in autophagic flux due to an increase in autophagosome synthesis. However, since most cells have a basal autophagic rate, autophagosome accumulation can also indicate a block of either autophagosome–lysosome fusion or the turnover of autolysosomes (29, 30). We therefore determined the cause of autophagosome accumulation in the absence of FUCA1. To assess this, we incubated wild-type and *Fuca1*-null MEFs with either bafilomycin A1 (BAF) or chloroquine (CQ), two agents that block the turnover stage of autophagy. In the context of these drugs, if loss of FUCA1 enhances autophagic flux, BAF or CQ treatment will cause an even greater accumulation of LC3-II in cells lacking FUCA1 when compared to controls. Conversely, if loss of FUCA1 causes autophagosome accumulation by impairing the turnover stage of autophagy, there should be no further accumulation of LC3-II in *Fuca1*-null cells when autophagy is also blocked by treatment with either BAF or CQ. Indeed, when this was tested, loss of FUCA1 caused an increase in LC3-II levels under baseline conditions, as previously observed, but FUCA1 loss did not cause any further accumulation of LC3-II beyond that observed upon treatment with either BAF or CQ, indicating that loss of FUCA1 causes LC3-II accumulation by impeding either the autophagosome-lysosome fusion or turnover stages of autophagy (Fig. 3*A*).

Although accumulation of LC3-II in *Fuca1*-null cells is indicative of a block in autophagosome/autolysosome turnover, it gives no readout of the true endpoint of autophagy: cargo digestion. To address this, we also assessed the impact of FUCA1 deletion in an assay we developed termed “enhanced mitophagy” (31). In this assay, cells are infected with parkin, a component of the E3 ubiquitin ligase complex required for mitochondrial turnover by autophagy. Previous studies have shown that cells ectopically expressing parkin undergo autophagy-dependent loss of mitochondria en masse following treatment with agents that perturb mitochondrial function (32, 33). We have found that this enhanced mitophagy can be used as a measure of autophagic cargo degradation, and therefore autophagic flux, because the rate and extent of loss of certain mitochondrial proteins is connected to the autophagic rate/capacity of the cell (31). Treatment of parkin-expressing cells with carbonyl cyanide m-chlorophenylhydrazone (CCCP) to damage mitochondria showed that mitochondrial proteins, in particular complex Va (CVa) and complex III core 1 (core 1), were markedly less degraded in *Fuca1*-null cells than in wild-type controls, demonstrating impaired autophagic flux and cargo digestion (Fig. 3*B*).

To measure the involvement of FUCA1 in response to a more natural autophagic stimulus, we analyzed wild-type and *Fuca1*-null cells under starvation conditions. To do this, we incubated cells in Earle’s balanced salt solution (EBSS), which lacks amino acids and thus is a potent inducer of autophagy (34). In this context, induction of autophagy leads to the “nonselective” degradation of cytoplasmic constituents, enabling us to determine whether the data we had obtained using enhanced mitophagy was a general or a mitochondria-specific effect. We examined the dynamics of autophagy following incubation in EBSS. During starvation, autophagosomes initially accumulate, as evidenced by an accumulation of LC3-II, but after a while cargoes are delivered to lysosomes, where they are degraded and their constituent parts recycled back into the cytoplasm. Since this degradation process involves the degradation of a portion of LC3-II, the levels of LC3-II decrease during this second stage (30). Moreover, the generation of increased levels of free amino acids from autophagic digestion leads to activation of mechanistic target of rapamycin complex 1 (mTORC1) and resultant inhibition of the initiation of autophagy (35). This effect further contributes to a reduction in the levels of LC3-II over time. Our analysis of this process in *Fuca1*-null cells revealed that LC3-II levels were higher at basal levels and accumulated in the initial period of starvation, and although the subsequent decrease in LC3-II levels was not entirely blocked, it was severely retarded when compared to controls (Fig. 3*C* and *SI Appendix*, Fig. S4 *B* and *C*). A similar effect was also observed by analysis of the levels of LC3^+^ puncta via immunofluorescence (*SI Appendix*, Fig. S4*D*). Collectively, these data suggest that FUCA1 contributes to successful autophagy, and that loss of FUCA1 and the changes in glycosylation that occur as a result greatly impair, but do not completely block, the turnover stage of the process.

Finally, to visualize the effects of FUCA1 in greater detail, we examined cells by electron microscopy following incubation in EBSS, which revealed accumulation of late autophagic vesicles containing undigested cargo in *Fuca1*-null cells (Fig. 3*D*). This again underscores the fact that FUCA1 contributes to successful autophagy, and that this effect relates to multiple cargoes.

### Changes in Glycosylation Caused by Loss of FUCA1 Affect Lysosomal Hydrolase Activity.

We were interested in understanding how loss of FUCA1 affects autophagic flux. Since autophagy is impaired at the turnover stage in the absence of FUCA1, but not completely blocked, we reasoned that loss of FUCA1 and associated glycosylation changes might affect the activity of proteins involved in this process. To identify proteins that might be affected by changes in glycosylation, we decided to take a SILAC (stable isotope labeling with amino acids in cell culture) mass spectrometry approach. We compared wild-type and *Fuca1*-null MEFs, and a Saos-2 cell line that is doxycycline-inducible for FUCA1 (TetOn-FUCA1). Cells from these FUCA1-low versus FUCA1-high comparisons were labeled with either “heavy” or “normal” amino acids. We then precipitated proteins from these cells using AAL because we reasoned that proteins with altered fucosylation due to loss or enhanced expression of FUCA1 would be precipitated to differing extents in the different FUCA1 contexts. This approach identified a number of proteins that were differentially precipitated by AAL in a FUCA1-dependent manner, including a number of lysosomal enzymes, including lysosomal α-glucosidase (GAA), glucosylceramidase (also referred to as β-glucosidase, GBA), β-hexosaminidase (HEXB), and α-mannosidase (MANBA) (*SI Appendix*, Table S2).

**Fig. 4.**
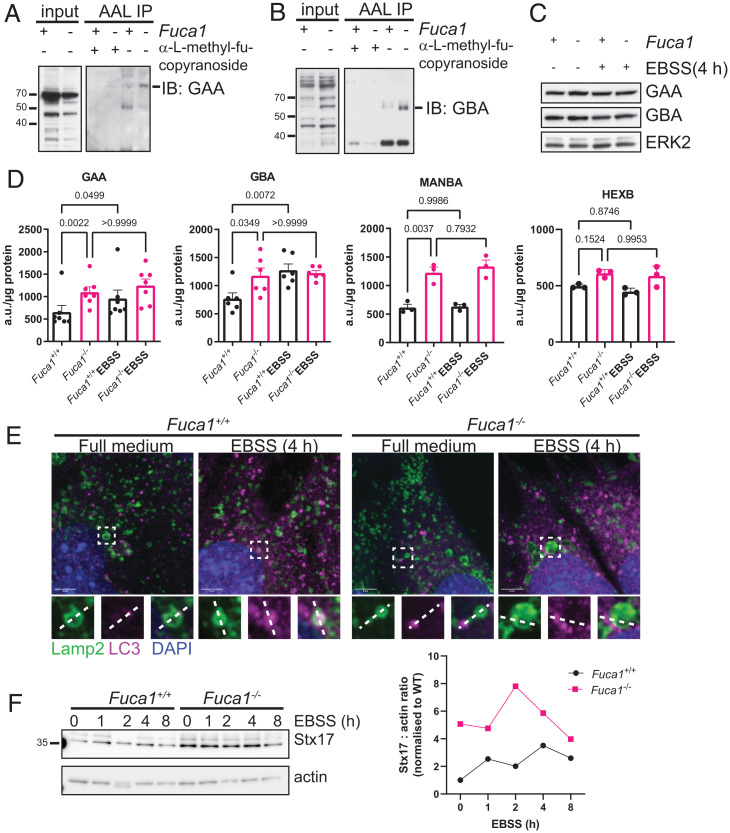
FUCA1 regulates fucosylation status and activities of lysosomal enzymes, and promotes autophagosome–lyosome fusion. (*A*–*D*) Primary *Fuca1*^flox/flox^ MEFs were infected with a retrovirus expressing the Cre recombinase (*Fuca1*^−/−^) or a retrovirus control empty vector (*Fuca1*^+/+^). Cell lysates were immunoprecipitated using biotinylated AAL, preincubated with methyl-α-l-fucopyranoside as a control (*A* and *B*). Eluted proteins were then loaded on a gel for immunoblotting analysis using antibodies directed against GAA (*A*) and GBA (*B*). Whole-cell lysates (input) were analyzed using the same antibodies. (*C*) Primary *Fuca1*^+/+^ and *Fuca1*^−/−^ MEFs were incubated in EBSS for 4 h. Cell lysates were analyzed by Western blot using antibodies against GAA, GBA, and ERK2, which was used as a loading control. (*D*) GAA, GBA, MANBA, and HEXB enzymatic activities were assessed in *Fuca1*^+/+^ and *Fuca1*^−/−^ MEFs under baseline and starvation conditions, and expressed as arbitrary units (a.u.) per microgram of total protein (*n* = 3 independent experiments, one-way ANOVA with Bonferroni multiple comparison test). (*E*) Subcellular localization of LC3B and Lamp2 was visualized in immortalized *Fuca1*^+/+^ and *Fuca1*^−/−^ MEFs under baseline conditions and after 4-h EBSS starvation by confocal microscopy. Nuclei were stained with DAPI. (Scale bars, 5 µm.) Regions of interest are highlighted in white boxes. (*F*) Immortalized *Fuca1*^+/+^ and *Fuca1*^−/−^ MEFs were incubated in EBSS for the indicated time period. Cell lysates were analyzed by Western blot using Stx17 and actin antibodies. The Stx17 : actin ratio is plotted on the right.

To confirm the results from our mass spectrometry screen and to further explore the lysosomal hydrolases that we identified, we performed pull-downs with AAL in wild-type and *Fuca1*-null MEFs. Precipitated lysates were then separated by Western blotting and probed with antibodies against GAA and GBA as examples. In each case, and in line with our SILAC results, the amounts of GAA and GBA pulled down in the absence of FUCA1 was higher than that in wild-type cells, indicating that FUCA1 modulates the glycosylation status of these important degradative enzymes (Fig. 4 *A* and *B*).

Although we identified lysosomal proteins whose glycan structure is changed by FUCA1, this does not mean they are connected to the decrease in autophagic flux caused by FUCA1 loss. To explore this possibility, we first examined the levels of GAA and GBA in wild-type and *Fuca1*-null MEFs under normal growth and amino acid-starved conditions. This revealed that the total levels of GAA and GBA were unaffected by loss of FUCA1 in either normal or amino acid-starved conditions (Fig. 4*C*). These results led us to consider whether the altered glycosylation state of these proteins may affect their enzymatic activities rather than their levels. In GAA, GBA, MANBA, and HEXB activity assays, we found that the activity of these enzymes was actually higher in the absence of FUCA1, which may represent a compensatory response to the decrease in autophagic flux (Fig. 4*D*) (19). However, when we looked at whether the activity of these proteins was induced in response to the stimulation of autophagy caused by starvation, we found that, like FUCA1 (*SI Appendix*, Fig. S5*A*), the activities of GAA and GBA were induced in wild-type MEFs, but further induction of GAA or GBA was not observed in MEFs lacking FUCA1 (Fig. 4*D*). These findings therefore suggest that upon amino acid deprivation, FUCA1 activity is induced and causes a change in the glycosylation status of GAA and GBA, which enhances the activity of these lysosomal enzymes. MANBA and HEXB activity, on the other hand, did not increase upon amino acid deprivation (Fig. 4*D*). Consequently, and perhaps through changes in other glycosylated proteins, FUCA1 promotes the adaptability of the hydrolytic capacity of lysosomes and facilitates autophagic flux.

### FUCA1 Loss Impairs Autophagosome–Lysosome Fusion.

Finally, we investigated whether the changes in autophagic flux under baseline and stress conditions (Fig. 3) in *Fuca1*-null cells were merely secondary to deregulated lysosomal activity as described above, or whether FUCA1 loss affects autophagy more directly. To this end we assessed a further upstream point of autophagy, namely autophagosome–lysosome fusion. We stained wild-type and *Fuca1*-null MEFs for Lamp2 and LC3 under basal and starvation conditions to visualize fusion events. We noticed that in wild-type cells, LC3 puncta were readily observed in the process of fusion with Lamp2^+^ vesicles, or even within those vesicles. In *Fuca1*-null cells, on the other hand, there were more instances of LC3 puncta adjacent to Lamp2^+^ lysosomes, suggesting that fusion events were less frequent (Fig. 4*E* and *SI Appendix*, Fig. S5*B*). To validate this observation, we measured the levels of the autophagosomal SNARE protein Stx17, which was more abundant in *Fuca1*-null cells both at baseline and during EBSS starvation (Fig. 4*F* and *SI Appendix*, Fig. S5 *C* and *D*). These findings suggest that loss of FUCA1 causes a stall in the fusion process, since Stx17 accumulation also occurs when the HOPS complex, which mediates autophagosome–lysosome fusion, is knocked down (36). Importantly, impairing lysosomal function in *Fuca1* wild-type cells by treatment with CQ alone did not lead to Stx17 accumulation (*SI Appendix*, Fig. S5*E*), indicating that FUCA1 affects autophagosome–lysosome fusion not only by causing a build-up of undegraded glycans in lysosomes. In summary, FUCA1 loss alters cellular homeostasis at multiple points by impairing lysosomal function, as well as deregulating autophagy at the turnover and autophagosome–lysosome fusion stages.

## Discussion

We report here that glycan breakdown, specifically defucosylation, is a contributing step in the process of macroautophagy. We show that loss of expression of the lysosomal glycosidase FUCA1 in cells and tissues causes accumulation of autophagosomes, indicating that FUCA1 modulates both basal and stimulated autophagy. Our further investigations revealed that FUCA1 supports both autophagosome–lysosome fusion and the turnover stage of autophagy, but that the absence of FUCA1 retards rather than completely blocks this process. It is notable that under starvation conditions, we found that FUCA1 loss decreases the rate of autophagosome clearance, as well as the degradation of mitochondria upon induction of mitophagy, indicating that FUCA1 is required for efficient autophagic flux in multiple contexts.

With hindsight from these studies, since at least 50% of all proteins are glycosylated (37, 38), it makes perfect sense that deglycosylation would be a facet of successful macroautophagy. We specifically chose to investigate FUCA1 due to its critical role in the removal of “core fucose”-linked α-1,6 to the *N*-acetylglucosamine, which is attached to asparagine in the peptide backbone (*N*-glycans). Previous reports have shown that without core fucose removal, further glycan breakdown is not possible (12, 15, 16, 39, 40). As a result, cells accumulate a variety of branched glycan species in their lysosomes. We considered—since it has been reported for mice lacking a functional mannose 6-phosphate receptor (41), which is required for delivery of proteins to the lysosome—whether the accumulation of glycans may completely perturb lysosomal function in the absence of FUCA1. We found, however, that lysosomal numbers were only minimally affected by loss of FUCA1. This indicates that the phenotype we observe is not due to a retardation of general lysosomal function, but instead appears to be due to the inability to induce the activity of degradative enzymes within the lysosome during increased autophagic flux. Due to the central role of FUCA1 in glycan breakdown, this naturally raises the question of whether mice deficient in other enzymes required for glycan breakdown, such as those that have specific roles in removing mannose or sialic acid, would also have retarded macroautophagy, and whether these glycosidases also act by perturbing the activity or inducibility of lysosomal hydrolases. In other words, is what we report here part of a process, which represents the autophagic breakdown of multiple forms glycans or does this only relate to breakdown of fucose-linked glycans in a more selective process? These questions, although beyond the scope of this report, are undoubtedly interesting and worthy of future investigation.

Although the principal aim of our study was to understand the importance of glycan breakdown in macroautophagy, our study also yields significant insights regarding the etiology of fucosidosis. Behavioral characterization of *Fuca1*^−/−^ mice revealed deficiencies in motor function and reduced exploration over time (habituation), characteristics consistent with a neurodegenerative phenotype as seen in fucosidosis patients and in a previous mouse model of the disease (20). Importantly we show, as has been reported for other lysosomal storage disorders, that the accumulation of lysosomal material in fucosidosis can be attributed to a defect in macroautophagy (42, 43). This raises the question of whether modulation of autophagy could be a reasonable approach for the treatment of fucosidosis. Since loss of FUCA1 does not block but retards autophagy flux, potent stimulation of autophagy might force accumulated lysosomal material through the bottleneck at the turnover stage caused by loss of FUCA1. This approach has been tried with other lysosomal storage disorders by blocking mTORC1 with rapamycin (44, 45). Alternatively, reducing the influx of material into the lysosome by reducing the initiation of autophagy may also be a way to reduce the cytotoxic accumulation of lysosomal material in both fucosidosis and other lysosomal storage disorders. This would, however, have to be done in a very measured way since total inhibition of autophagy initiation itself has significant detrimental effects. However, the prospect of emerging autophagy modulators combined with mouse models of fucosidosis and other lysosomal storage disorders may forge a path toward treating these rare, but often severe metabolic disorders.

## Materials and Methods

### *Fuca1* Conditional Knockout Generation.

To generate the conditional gene targeting vector for *Fuca1*, a 129 bacterial artificial chromosome (BAC) for the *Fuca1* genomic locus was initially identified on Ensembl (www.ensembl.org) (46). Three homology arms were subcloned from the BAC by recombineering in DY380 *Escherichia coli* according to standard protocols (47). The arms were then cloned into the targeting vector pFlexNeoDTA, a modified version of pFlexible (46) with a PGK-EM7-Neo cassette (from pL452) (47), replacing the puroΔtk and introducing a DTA-negative selection cassette (48). The targeting vector was linearized by restriction digestion and electroporated into HM1 mouse embryonic stem (ES) cells (49) and selected under G418 (240 µg/mL active reagent; Formedium, G418S). Surviving colonies were picked and screened for targeting by long-range PCR reactions using the Expand Long Template PCR System (Roche, 11681834001). Appropriate targeting was confirmed at both the 5′ and 3′ sides. The presence of the isolated loxP was confirmed by PCR across the site.

Following identification of correctly targeted clones, mouse lines were generated by microinjection of ES cells into C57BL/6J blastocysts. After breeding of chimeras, germline offspring were identified by coat color, and the presence of the modified allele was confirmed by PCR. Mice were subsequently crossed with the Del cre B6.C-Tg(CMV-cre)1Cgn/J-mouse line (Jackson Laboratory) to delete *Fuca1* in the whole body (50).

### Mouse Maintenance.

All animal procedures were approved by the Ethical Review Process of The University of Glasgow and conducted in compliance with UK Home Office Animal (Scientific Procedures) Act 1986, including Amendment Regulations 2012 (project license number: P54E3DD25). No blinding was applied during experiment analysis.

### Behavioral Tests.

Female and male *Fuca1*^+/+^, *Fuca1*^−/−^, and *Fuca1*^+/−^ littermates aged 180 d ± 20 d (*n* = 10 for each group) were submitted to a SHIRPA test battery. Additional behavioral testing included the hanging wire and open-field tests.

During the hanging-wire test, the mouse was placed onto a wire cage lid; the lid was then shaken three times to encourage the mouse to grip onto the wire. The cage lid was then inverted and the latency to fall off was measured. Each mouse was given three trials and the average time on the wire was calculated.

For the open-field test, a 30-min assessment of exploratory behavior and motor output when placed in a novel environment was conducted. The open field arena was 40 × 40 × 40 cm. All animal movements were tracked using the Noldus Ethovision tracking software package (v8.5). The main measures taken were distance traveled and velocity.

All data were analyzed using univariate ANOVA with fixed factors of genotype and sex. Where appropriate, restricted analysis was performed using one-way ANOVA and for measures with time bins, a general linear model repeated ANOVA was used, within-subjects factors of time bin, and between-subjects factors of genotype and sex.

### Cell Culture, MEF Isolation, Treatment, and Retroviral and Lentiviral Infection.

All cell lines were cultured in DMEM (Gibco, 21696-035) containing 10% FBS, 4.5 g L^−1^ glucose, 1 mM l-glutamine, 0.11 g L^−1^ pyruvate, and were maintained at 37 °C in 5% CO_2_ atmosphere and were routinely tested for mycoplasma.

*Fuca1*^flox/flox^ MEFs were isolated from embryonic day 13.5 embryos. Where stated, immortalized *Fuca1*^flox/flox^ MEFs were used. To immortalize MEFs, primary *Fuca1*^flox/flox^ MEFs were passaged 1:4 every 3 d until they entered senescence. After 2 mo, surviving *Fuca1*^flox/flox^ MEFs were established into an immortalized line.

To generate *Fuca1* knockout MEFs, *Fuca1*^flox/flox^ MEFs were retrovirally infected with pBabe-puro-Cre or a retroviral vector control (51). Briefly, Phoenix Eco packaging cells were transfected with pBabe-puro-Cre or pBabe-puro empty vector by Ca_3_(PO_4_)_2_ precipitation. Retroviral supernatants were added to *Fuca1*^flox/flox^ MEFs together with 10 µg/mL polybrene (hexadimethrine bromide; Sigma, H9268) every 12 h. After the third round of infection, cells were selected for 5 d in 2.5 µg/mL puromycin (Sigma, P9620). To induce autophagy, cells were washed in PBS three times and then incubated for the indicated amount of time in EBSS (Sigma, E2888). In order to inhibit autophagy, cells were incubated in 100 nM Bafilomycin A1 (Bio-Techne, 1334) or 10 µM hydroxychloroquine (Sigma, C6628) for 4 h.

TetOn-FUCA1 Saos-2 cells were generated by transfection with pTRE-FUCA1 and pIRES-Hyg-EcoR followed by clonal selection in 100 μg/mL hygromycin (Gibco, 10687010). pTRE-FUCA1-Myc/His was generated through digestion of pcDNA3-FUCA1-Myc/His with HindIII and Pme1 followed by cloning in to the HindIII and EcoRV sites of pTRE2 (Clontech-Takara Bio, 6241-1). Expression of FUCA1 was induced with 1 mg/mL doxycycline (Sigma, D9891) for 48 h.

### qRT-PCR.

qRT-PCR analysis was undertaken as previously described (52). *Fuca1* primers are QuantiTect primers from Qiagen (QT00053802). All samples were normalized to 18S ribosomal RNA and expressed as *Fuca1* relative mRNA expression.

### Histology and Immunohistochemistry.

Immediately after culling, mouse tissues were fixed for at least 24 h in 10% neutral buffered formalin (Sigma, HT501128). Tissue sections (4 µm) were cut from paraffin-embedded blocks and stained with hematoxylin (Cell Path, RBA-4201-00A) and eosin (VWR, 341973R), or LC3B antibody (0231-100, Nanotools), or VDAC antibody (Cell Signaling Technology, #4866) as previously described (53). For the lectin staining, tissue sections were dewaxed in xylene for 5 min followed by rehydration through pure ethanol (2 × 1 min) then 70% ethanol (1 × 1 min). The sections were rinsed in water, then endogenous peroxidase was blocked using Dako Peroxidase block (Agilent, S2023) for 5 min. The sections were rinsed in PBS before the biotinylated lectin was applied at a predetermined optimal dilution (biotinylated AAL and UEA1; 1:2,000) (Vector Labs, B-1395 and B-1065). The lectin was applied for 30 min, then the sections were rinsed in PBS/Tween (2 × 4 min) and had VECTASTAIN Elite ABC-HRP kit (Vector Labs, PK-6100) applied for 30 min as per manufacturer’s instructions. The sections were rinsed in PBS/Tween (2 × 4 min) and then the visualization substrate (3,3′-Diaminobenzidine tetrahydrochloride; Agilent, K3468) was applied for 5 min. This reaction was terminated in water.

### Pathology Analysis.

H&E- and lectin-stained tissue sections were reviewed by two pathologists. The presence or absence of cytoplasmic vacuolation and glycan accumulation in tissues (with specification of the affected cell types) was assessed by consensus in all examined tissues.

### Immunofluorescence.

Cells were seeded on coverslips overnight, and then fixed in 4% paraformaldehyde (CN Tech, 15710) for 20 min, washed in PBS once, then permeabilized in 0.5% Triton X-100 (Sigma, T9284) or 0.1% saponin (Sigma, 84510) in PBS for 15 min. Immunofluorescence staining was performed as previously described (54) using a rabbit anti-LC3B antibody (Cell Signaling Technology, #2775) and a rat anti-LAMP2 (EMD Millipore, MABC24). Secondary antibodies included Alexa Fluor 488 anti-rabbit IgG (ThermoFisher Scientific, A11008), Texas red anti-rat IgG (ThermoFisher Scientific, T6392), Alexa Fluor 555 anti-rabbit IgG (ThermoFisher Scientific, A21429), Alexa Fluor 488 anti-rat IgG (ThermoFisher Scientific, A11006), and were incubated with 5 µg/mL DAPI (Sigma, D9542). Imaging was carried out using the Zeiss 710 confocal microscope using the 63× objective. Total volume of Lamp2^+^ and LC3^+^ compartments were quantified from maximum-intensity projection images obtained from *z*-stack images using Volocity 3D Image Analysis Software, and expressed as the sum of intensity per cell (Fig. 2*D*). To plot the line graphs representing relative localization of Lamp2 and LC3 (*SI Appendix*, Fig. S5*B*), we used the “Intensity” macro in ImageJ, previously developed by Imperial College London (https://www.imperial.ac.uk/medicine/facility-for-imaging-by-light-microscopy/software/fiji/).

### Lysotracker.

Cells were stained with 1 µM LysoTracker Red DND-99 (ThermoFisher Scientific, L7528) for 2 h at 37 °C. Staining was analyzed by flow cytometry using a FACS Calibur.

### DQ-BSA and Dextran Time Course.

Immortalized *Fuca1*^+/+^ and *Fuca1*^−/−^ MEFs, no more than five passages after retroviral infection, were plated in six-well plates. The next day, cells were washed in PBS twice before incubation in EBSS for 1 h. After 1 h, the EBSS was replaced with EBSS containing 10 µg/mL DQ-green-BSA (ThermoFisher Scientific, D12050) and 20 µg/mL anionic, fixable AF647-labeled dextran (ThermoFisher Scientific, D22914) for the indicated time. At the last time point, cells were washed twice with ice-cold PBS to remove excess dyes. Cells were fixed using BD CytoFix buffer (BD Biosciences, AB_2869005) for 15 min on ice. Flow cytometry was performed on a BD LSR Fortessa (BD Biosciences) and data were analyzed using FlowJo software.

### Transmission Electron Microscopy.

Samples were prepared by standard procedures as previously described (55). Briefly, cell pellets were fixed overnight in 2.5% glutaraldehyde in 0.1 M sodium phosphate buffer at 4 °C. The pellets were then divided and incubated for 1 h at room temperature in 1% osmium in 100 mM cacodylate buffer. Cells were then dehydrated in increasing concentrations of ethanol and embedded in Epon resin. Ultrathin sections were prepared on copper grids, stained with uranyl acetate and lead citrate. Ultrastructures were imaged using JEOL 1400 plus electron microscope and an AMT UltraVUE camera.

### Western Blotting.

Cells were lysed in buffer containing 1% Triton X-100, 0.1% SDS, 50 mM Hepes pH 7.5, 150 mM NaCl, 100 mM NaF, 10 mM EDTA, 10 mM Na_4_P_2_O_7_ and protease inhibitors (Roche, 04693124001) as previously described (31). Protein concentrations were determined by BCA assay (Pierce Thermo Scientific, 23225) or DC assay (Bio-Rad, #5000116). Cell lysates were separated by SDS/PAGE and transferred either by wet transfer onto Immobilon-P membranes (Merck, IPVH00010) or by semidry transfer onto Trans-Blot Turbo RTA Mini 0.2-µm PVDF membranes (Bio-Rad, #1704272). Membranes were probed with LC3B (Cell Signaling Technology, #2775), GAA (Abcam, ab137068), GBA (Abcam, ab92997), ERK2 (Santa Cruz Biotechnology, sc-154), p62 (Enzo Life Sciences, BML-PW9860), Stx17 (ThermoFisher Scientific, #PA5-40127), and β-actin (Abcam, ab8227) antibodies. To assess mitochondrial protein loss, membranes were probed with MitoProfileMembrane Integrity WB Antibody mixture (Abcam, ab110414).

### Enhanced Mitophagy Assay.

To generate *Fuca1*^flox/flox^ LZRS-YFP-PARKIN cells, immortalized *Fuca1*^flox/flox^ MEFs were retrovirally infected using pLZRS-YFP-Parkin, as previously described (31, 33).

Mitochondrial depletion was triggered by treatment with 12.5 µM CCCP (Sigma, C2759) two times in 48 h, as previously described (31, 33). Following treatments, all floating cells were discarded and cell lysates were analyzed by Western blotting.

### SILAC Labeling.

TetOn-FUCA1 Saos-2 cells and immortalized *Fuca1*^flox/flox^ MEFs were cultured in DMEM containing light amino acids (Arg0/Lys0) or heavy amino acids (Arg10/Lys8) (Cambridge Isotopes Laboratories: A6969, A6969, CNLM-539-H-1, and CNLM-291-H-1) supplemented with 10% dialyzed FBS until more than 99% of heavy amino acids were incorporated. Cells were subsequently treated with doxycycline (Sigma, D9891) or retrovirally infected with pBabe-puro-Cre or a retroviral control vector. Cells were lysed and equal amounts of light and heavy SILAC-labeled lysates (500 µg) were mixed prior to immunoprecipitation.

### Immunoprecipitation.

For immunoprecipitation, 500 µg of lysates were incubated with 20 µg of biotinylated AAL (Vector Labs, B-1395-1) overnight at 4 °C. As a negative control, AAL was preincubated with 200 mM methyl-α-l-fucopyranoside (Carbosynth, MM02387) for 1 h prior to incubation with the lysate. Next, 100 µL of Dynabeads MyOne Streptavidin T1 (ThermoFisher Scientific, 65601) were then added for 1 h at 4 °C. After washing in PBS 0.01% Tween four times, fucosylated proteins were eluted by incubating the beads with 200 mM of methyl-α-l-fucopyranoside for 2 h at 20 °C. Proteins were then loaded on a gel for mass spectrometry analysis or Western blot analysis.

### Lysosomal Enzyme Assays.

The enzymatic activity of α-l-fucosidase was assessed as previously described (56). α-Glucosidase and β-glucocerebrosidase activities were also assessed using modified versions of this previously described protocol. Briefly, cells were lysed in a buffer containing 1% TTX, 0.1% SDS, 50 mM Hepes pH 7.5, 150 mM NaCl, 100 mM NaF, 10 mM EDTA, 10 mM Na_4_P_2_O_7_ and protease inhibitors (Roche, 04693124001), and the protein concentrations were determined by BCA assay (Pierce Thermo Scientific, 23225). Subsequently, 15 μg of protein (diluted in 0.2 M sodium acetate buffer to a volume of 100 μL) was incubated with 100 μL of 0.2 mM 4-methylumbelliferyl α-l-fucopyranoside (Sigma, M8527), 0.2 mM 4-methylumbelliferyl α-d-glucopyranoside (Sigma, 69591), or 0.2 mM 4-methylumbelliferyl β-d-glucopyranoside (Sigma, M3633), 0.2 mM 4-methylumbelliferyl α-d-mannopyranoside (Sigma, 69694), substrates for α-l-fucosidase, α-glucosidase, β-glucocerebrosidase, and α-mannosidase, respectively, in a 96-well plate. The plate was incubated at 37 °C for 90 min and 4-methylumbelliferone fluorescence was measured using a plate reader (excitation 365 nm/emission 450 nm). β-Hexosaminidase activity was measured by lysing 500,000 cells in 50 µL of 0.1% Triton X-100 in MilliQ water containing protease inhibitors (Roche, 04693124001). Subsequently, 15 µL of this lysate was incubated with 15 µL 1 mM 4-nitrophenyl *N*-acetyl-β-d-glucosaminide (Sigma, N9376) in a 96-well plate. The plate was incubated at 37 °C for 60 min and the reaction was quenched by addition of 250 µL 0.1 M carbonate/bicarbonate buffer (Sigma, C3041). Absorbance was measured at 405 nm using a plate reader (57).

### Mass Spectrometry Acquisition.

Each gel lane was excised into nine slices, which were reduced using 10 mM dithiothreitol (Sigma, 43815), alkylated with 55 mM iodoacetamide (Sigma, I1149), and digested with trypsin (Trypsin gold, Promega, V5280) for 12 h at 30 °C. Tryptic peptides were dried in a centrifugal evaporator (ThermoFisher Scientific, SPD131DDA). Tryptic peptides were analyzed on a Linear Trap Quadrupole (LTQ)-Orbitrap Velos (ThermoFisher Scientific) coupled online to an EASY-nLC II (ThermoFisher Scientific). Dried tryptic peptides were resuspended in and loaded with buffer A (2% acetonitrile [Rathburn, RH1016/1], 0.1% formic acid [Merck, 100264]) on a precolumn NS-MP-10dp 1.9 μm, 360/100 μm × 0.2 cm (NanoSeparations), and washed with 25 µL of buffer A at constant pressure of 200 bar.

Peptides were then separated on a 20-cm fused silica emitter (New Objective FS360-75-8-N-5-C20 40) packed in-house with ReproSil-Pur C18-AQ 1.9-μm resin (Dr Maisch GmbH) using a flow of 200 nL/min in a 42-min gradient from 5 to 28% buffer B (80% acetonitrile, 0.1% formic acid), followed by 13-min gradient from 28 to 45% buffer B. The emitter was kept at 36 °C with a column oven integrated into the nanoelectrospray ion source (Sonation PRSO-V1). An Active Background Ion Reduction Device (ABIRD, ESI Source Solutions) was used to decrease contaminant signal level. The MS spectra were acquired in the Orbitrap analyzer at a resolution of 60,000 at 400 *m*/*z*, and a target value of 106 charges. Collision induced dissociation fragmentation of the 10 most intense ions was performed using a target value of 5,000 charges and recorded in the LTQ. Data were acquired with XCalibur software.

### Mass Spectrometry Data Analysis.

Raw data obtained were processed with the MaxQuant software v1.5.2.8 and searched with Andromeda search engine, querying SwissProt *Homo sapiens* (55,761 entries) and *Mus musculus* (20,273 entries) databases. Protein hits coming from each individual database were separated using the “Split protein groups by taxonomy ID” option, which is available in MaxQuant. The first and main searches were performed with precursor mass tolerances of 20 ppm and 4.5 ppm, respectively, and MS/MS tolerance of 0.5 Da. The minimum peptide length was set to 6 amino acids and strict specificity for trypsin cleavage was required, allowing up to two missed cleavage sites. Carbamidomethylation (Cys) was set as fixed modification, and oxidation (Met) and *N*-acetylation were specified as variable modifications. The peptide, protein, and site false-discovery rate was set to 1%. At least one uniquely assigned peptide and a minimum ration count of 2 were required for a protein to be quantified. The relative quantification of the peptides against their SILAC-labeled counterpart was performed by MaxQuant. Only protein groups quantified in both forward and reverse experiment are reported in the results.

## Supplementary Material

Supplementary File

## Data Availability

All data have been deposited in a publicly accessible database called Enlighten: Research Data (https://doi.org/10.5525/gla.researchdata.1280) (58).
