# Supplementary material for: Glycan degradation promotes macroautophagy
Source: Proc Natl Acad Sci U S A. 2022 Jun 22;119(26):e2111506119. doi: 10.1073/pnas.2111506119 (PMC9245654; doi:10.1073/pnas.2111506119)
Supplement: Supplementary File [file pnas.2111506119.sapp.pdf]

Supplementary Figure Legends:

Figure S1: Histological analysis of glycosylated species in FUCA1-null mice.

(A) Sections from the cerebellum, brain cortex, pancreas, liver and kidney derived from both *Fuca1*$^{+/+}$ (upper panel), *Fuca1*$^{-/-}$ (lower panel) mice were stained with *Ulex Europaeus* agglutinin I *(*UEAI). Red arrows indicate strong UEA1 staining in the pancreas. The images shown are representative of changes observed in 6 mice (aged between 90 and 220 days). Pictures were taken using a Zeiss AX10 microscope with a 40x objective. Scale bar, 20 µm.

Figure S2: Phenotypic analysis of mouse fucosidosis model.

(A) Kaplan-Meier analysis of *Fuca1*$^{+/+}$ (n = 22), *Fuca1*$^{+/-}$ (n = 28), *Fuca1*$^{-/-}$ (n = 33) mice (Log-rank (Mantel-Cox) test ***p<0.0001). Mice sacrificed before endpoint due to phenotype-unrelated circumstances were censored from the study (indicated as tick marks in the Kaplan-Meier curve). (B) Body weight in grams (g) of *Fuca1*$^{+/+}$, *Fuca1*$^{+/-}$, *Fuca1*$^{-/-}$ female (upper panel; n = 9 per genotype, mean ± SEM) and male (lower panel; n = 10 per genotype, mean ± SEM) littermates over a period of 34 weeks (two-way ANOVA, Tukey's multiple comparison test; *Fuca1*$^{+/+}$ versus *Fuca1*$^{-/-}$ *p<0.05; **p<0.01*; Fuca1*$^{+/+}$ versus *Fuca1*$^{+/-}$ $^{\$}$p<0.05). (C) Liver weight of *Fuca1*$^{+/+}$ (n = 12), *Fuca1*$^{+/-}$ (n = 9), *Fuca1*$^{-/-}$ (n = 11) adult females aged between 90-257 days (upper panel, mean ± SEM) and *Fuca1*$^{+/+}$ (n = 9), *Fuca1*$^{+/-}$ (n = 6), *Fuca1*$^{-/-}$ (n = 8) adults males aged between 90-227 days (lower panel, mean ± SEM) (Kruskal-Wallis test **p=0.0002). (D) Average time spent on the wire (over 3 sessions) for the 3 groups (n = 10 per genotype per sex). Data are represented as mean ± SEM (post-hoc analysis of genotype with Bonferroni correction; *p=0.011). (E) Distance travelled in the open field for both female (upper panel) and male (lower panel) mice

for all 3 genotypes (n = 10 per genotype per sex). Data are shown as mean ± SEM (post-hoc analysis with Bonferroni correction; *Fuca1*$^{-/-}$ versus *Fuca1*$^{+/+}$ *p = 0.001; *Fuca1*$^{-/-}$ versus *Fuca1*$^{+/-}$ **p = 0.0001; *Fuca1*$^{+/+}$ versus *Fuca1*$^{+/-}$ p = 1.00) . (F) Velocity in the open field for both male and female mice for all 3 genotypes (female upper panel, male lower panel; n = 10 per genotype per sex). Data are represented as mean ± SEM (post-hoc analysis with Bonferroni correction; *Fuca1*$^{-/-}$ versus *Fuca1*$^{+/+}$ *p = 0.001; *Fuca1*$^{-/-}$ versus *Fuca1*$^{+/-}$ **p = 0.0001; *Fuca1*$^{+/+}$ versus *Fuca1*$^{+/-}$ p = 1.00).

Figure S3: VDAC staining in *Fuca1*$^{+/+}$ and *Fuca1*$^{-/-}$ brains.

(A) Sections from the various regions of the brain derived from both *Fuca1*$^{+/+}$ (upper panel) and *Fuca1*$^{-/-}$ (lower panel) mice were stained for the mitochondrial protein VDAC. Red arrows indicate VDAC accumulation. The images shown are representative of changes observed in 5 mice per genotype (aged between 90 and 100 days). Pictures were taken using a Zeiss AX10 microscope with a 40x objective. Scale bar, 20 μm.

Figure S4: FUCA1 loss does not affect general endo-lysosomal trafficking, but impairs autophagy flux.

(A) Flow cytometric time course analysis of DQ-BSA and dextran fluorescence in immortalised *Fuca1*$^{+/+}$ and *Fuca1*$^{-/-}$ MEFs. Median fluorescence intensity was adjusted to 0 at the 0 hour time point. Data are represented as mean ± standard deviation (n = 3 independent MEF lines). (B) and (C) represent independent repeats of the experiment shown in Figure 3C: Immortalised *Fuca1*$^{+/+}$ and *Fuca1*$^{-/-}$ MEFs were incubated in EBSS for the indicated time period. Cell lysates were analysed by Western blot using LC3B and actin antibodies. The LC3II : actin ratio is plotted on

the right. (D) LC3B and Lamp2 were visualised in *Fuca1*$^{+/+}$ and *Fuca1*$^{-/-}$ MEFs after EBSS starvation by confocal microscopy using an anti-LC3B antibody and anti-LAMP2 antibody. Nuclei were stained with DAPI. Scale bar, 20 µm.

Figure S5: FUCA1 contributes to autophagosome-lysosome fusion.

(A) FUCA1 enzymatic activity was assessed in *Fuca1*$^{+/+}$ and *Fuca1*$^{-/-}$ MEFs under baseline and starvation conditions, and expressed as arbitrary units (a.u.) per µg of total protein (n = 7 independent experiments, one-way ANOVA with Bonferroni multiple comparison test). (B) The high-power immunofluorescence images from Figure 4E are depicted here with line graphs showing the relative localisation of Lamp2-positive structures and LC3 puncta. These data are representative of at least four fields of view. (C) and (D) represent independent repeats of the experiment shown in Figure 4F: Immortalised *Fuca1*$^{+/+}$ and *Fuca1*$^{-/-}$ MEFs were incubated in EBSS for the indicated time period. Cell lysates were analysed by Western blot using Stx17 and actin antibodies. The Stx17 : actin ratio is plotted on the right. (E) Immortalised *Fuca1*$^{+/+}$ MEFs were treated with 10 µM hydroxychloroquine for 4 hours. Cell lysates were analysed by Western blot using antibodies against Stx17 and actin, which was used as a loading control. Two experiments are shown side by side.

A

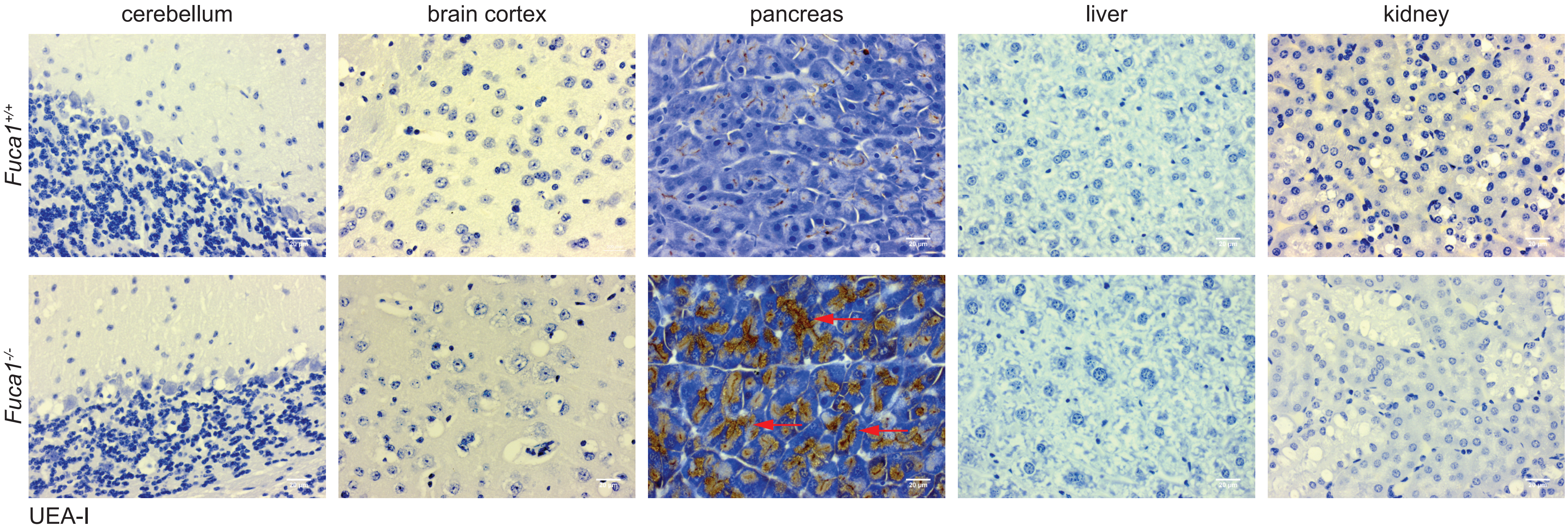

Baudot_FigS1

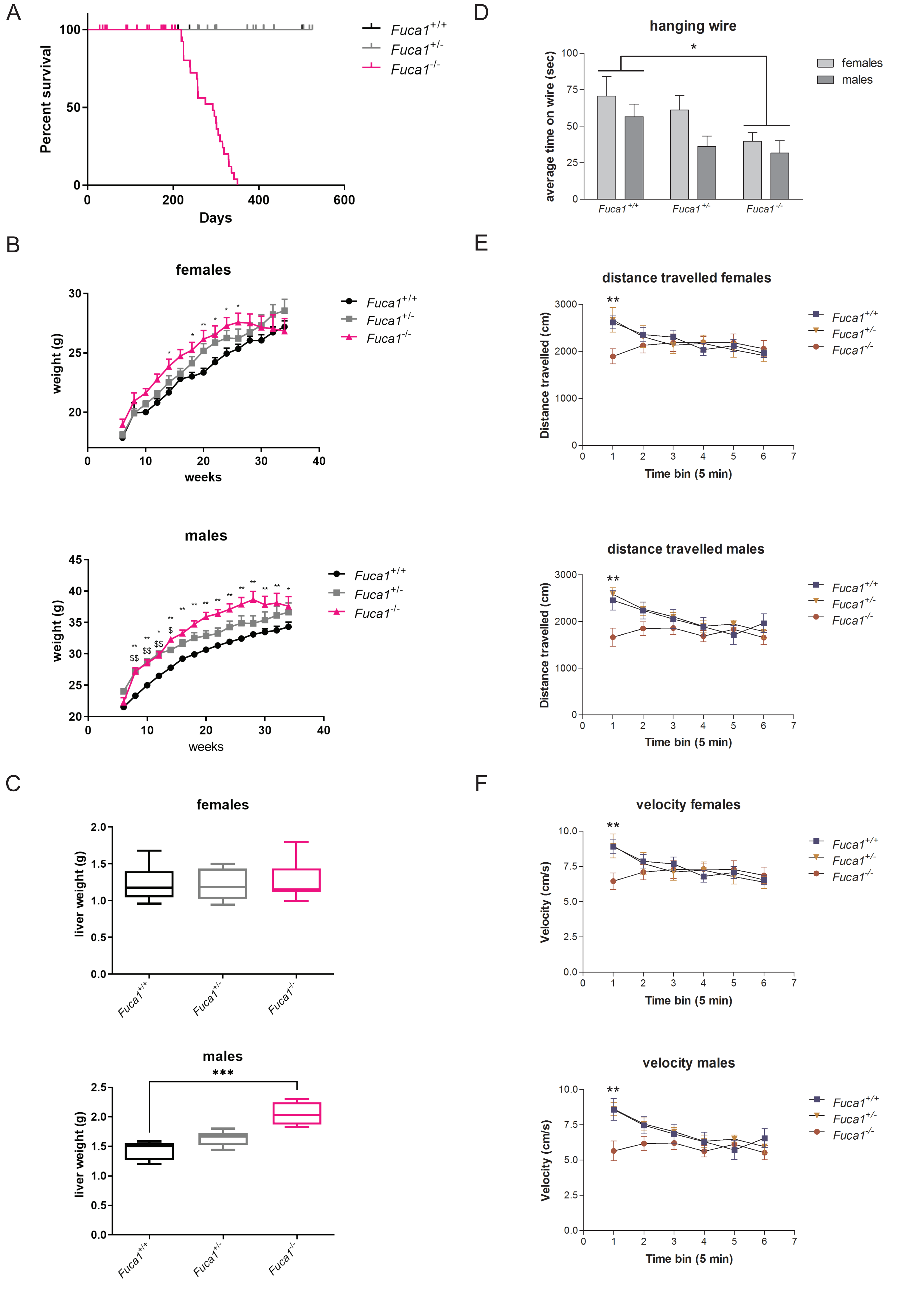

Baudot_FigS2

A

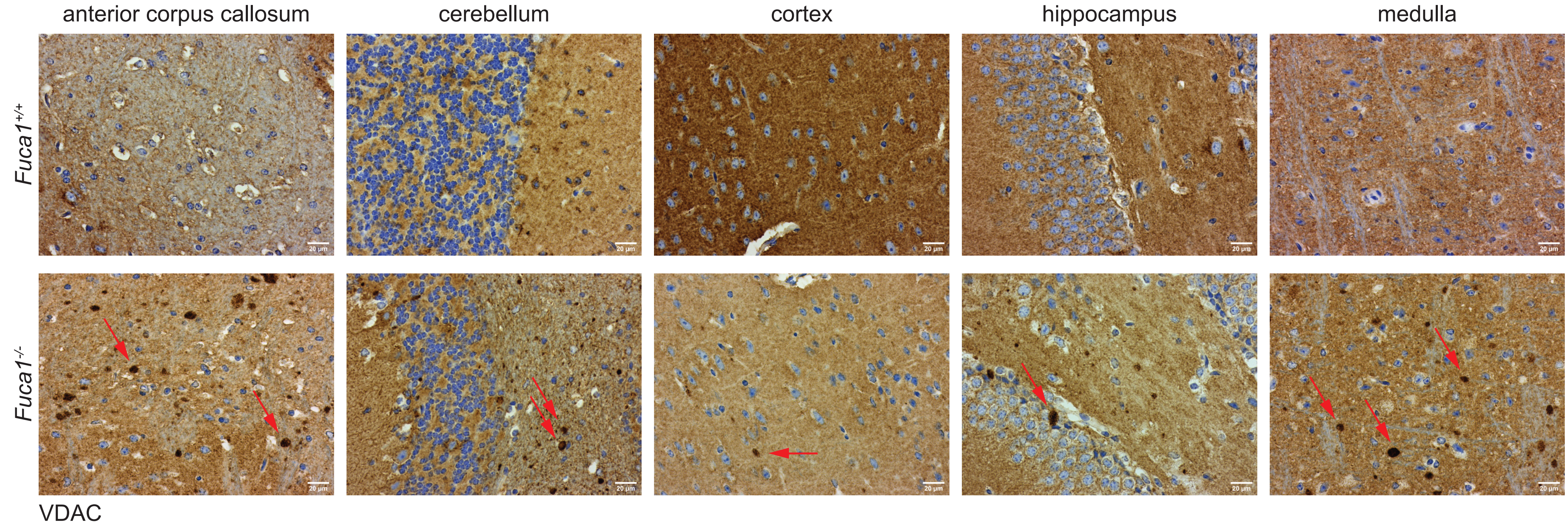

Baudot_FigS3

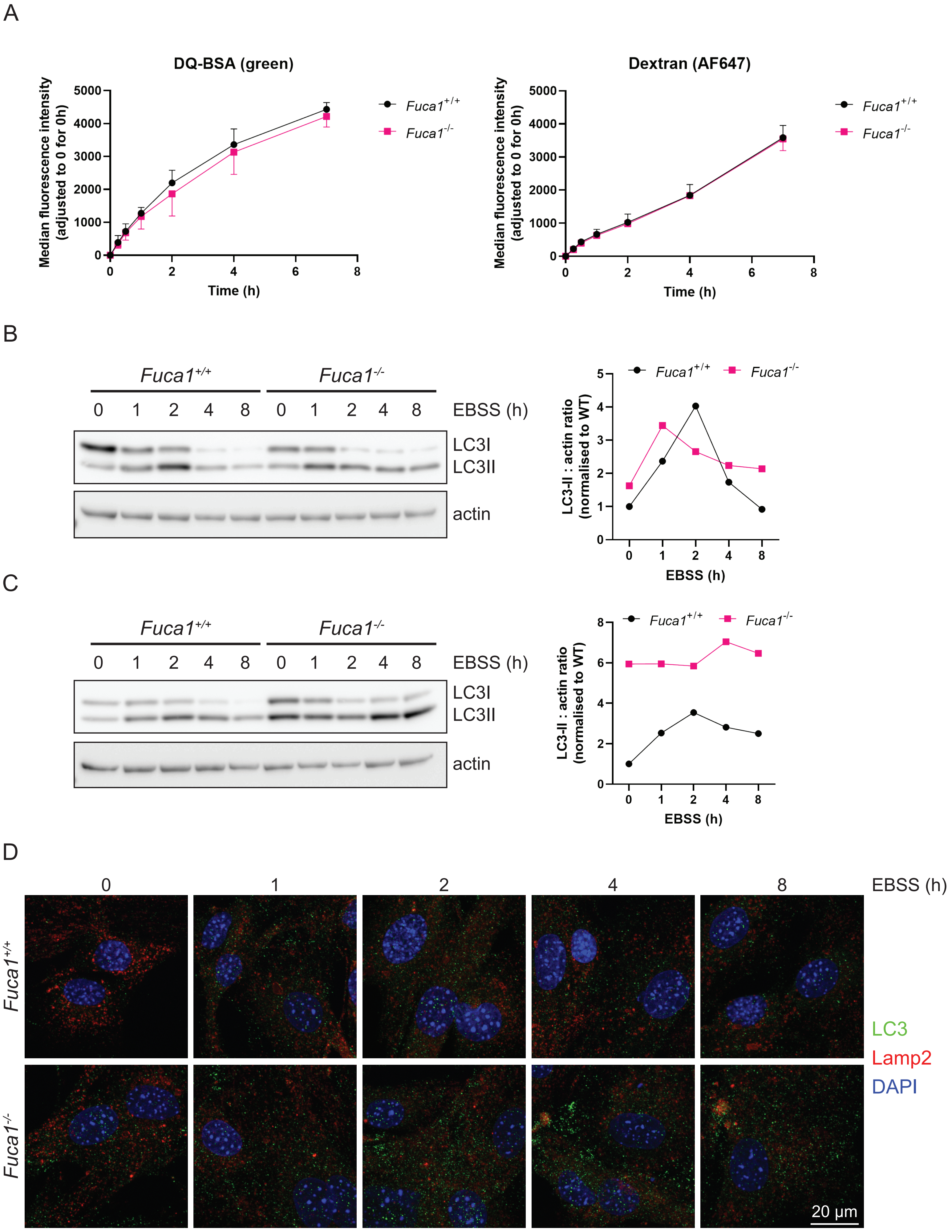

Baudot_FigS4

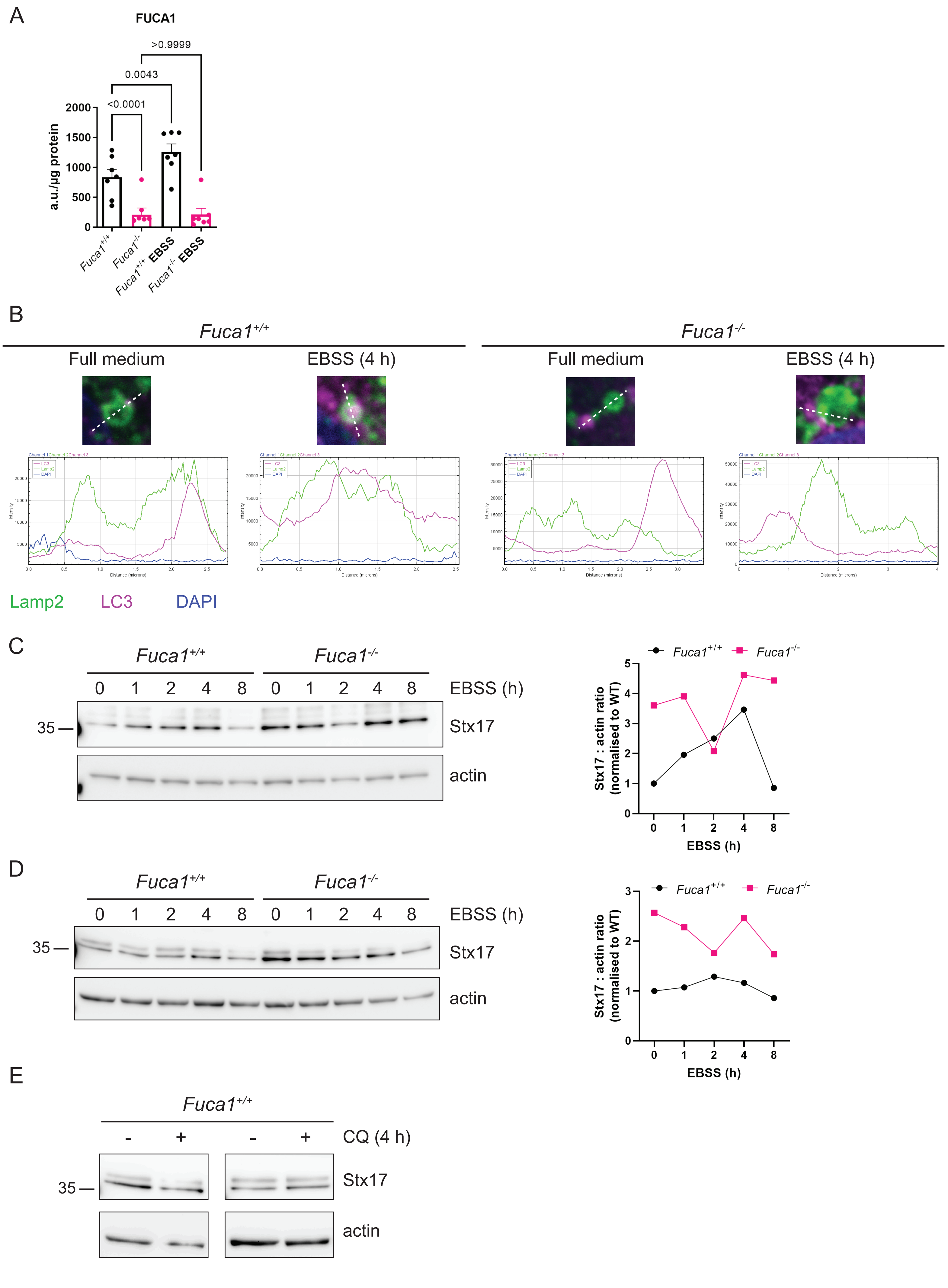

Baudot_FigS5

measure	Male Fuca+/+	Male Fuca$^{+/-}$	Male Fuca$^{-/-}$	Female Fuca$^{+/+}$	Female Fuca$^{+/-}$	Female Fuca$^{-/-}$
body position	3	3	3	3	3	3
spontaneous activity	2	2	2	2	2	2
tremor	N	N	N	N	N	N
urination?	Y	Y	Y	Y	Y	Y
defecation?	Y	Y	Y	Y	Y	Y
bizzare behaviours	N	N	N	N	N	N
convulsions	N	N	N	N	N	N
palebral closure	EYES OPEN	EYES OPEN	EYES OPEN	EYES OPEN	EYES OPEN	EYES OPEN
piloerection	N	N	N	N	N	N
gait	Normal and fluid	Normal and fluid	Normal and fluid	Normal and fluid	Normal and fluid	Normal and fluid
pelvic elevation	Normal	Normal	Normal	Normal	Normal	Normal
tail elevation	Horizontal and extended	Horizontal and extended	Horizontal and extended	Horizontal and extended	Horizontal and extended	Horizontal and extended
pinna reflex	Active	Active	Active	Active	Active	Active
cornea reflex	Active	Active	Active	Active	Active	Active
lacrimation	N	N	N	N	N	N
provoked biting	Y	Y	Y	Y	Y	Y
trunk curl	Y	Y	Y	Y	Y	Y
visual placing	Upon whisker contact	Upon whisker contact	Upon whisker contact	Upon whisker contact	Upon whisker contact	Upon whisker contact

Table S1: *Phenotypic assessment of Fuca1$^{+/+}$, Fuca1$^{+/-}$ and Fuca1$^{-/-}$ mice in elements of the SHIRPA test battery.*

Protein names	Gene names	Average Ratio norm. (-)FUCA1/(+)FUCA1 (f/r)	Average Ratio norm. CRE/CTRL (f/r)
Lysosomal Pro-X carboxypeptidase	PRCP	2.20985	1.57206
Cathepsin B	CTSB	2.08228	1.86527
Bis(5-adenosyl)-triphosphatase	ENPP4	1.82044	1.68005
Epididymis-specific alpha-mannosidase	MAN2B2	1.55831	1.59851
Glucosylceramidase	GBA	1.51113	1.29402
Lysosomal alpha-glucosidase	GAA	1.39682	1.41766
Putative phospholipase B-like	PLBD2	1.35757	1.3653
Beta-hexosaminidase subunit beta	HEXB	1.21077	1.72529
Lysosomal acid phosphatase	ACP2	1.15862	1.43828
Carboxypeptidase Q	CPQ	1.14963	1.96311
Beta-galactosidase	GLB1	1.14068	1.34898
N-acetylglucosamine-6-sulfatase	GNS	1.12606	1.62317
Alpha-N-acetylglucosaminidase (Sanfilippo disease IIIB)	NAGLU	1.11974	1.54377
N-sulphoglucosamine sulphohydrolase	SGSH	1.08221	1.65267
Alpha-galactosidase A	GLA	1.07791	1.6124
Hepatocyte growth factor receptor	MET	1.05726	1.50077
Carbohydrate sulfotransferase 14	CHST14	1.0399	1.34604
Lysosomal alpha-mannosidase	MAN2B1	1.03948	2.09151
Integrin alpha-5	ITGA5	1.01885	1.29505
Polypeptide N-acetylgalactosaminyltransferase 10	GALNT10	0.989992	1.67479
Cell surface glycoprotein MUC18	MCAM	0.958456	2.10877

Baudot_Supplementary Table 2: *Proteins exhibiting differential fucosylation upon over-expression or deletion of FUCA1*
